# Observations on the Letter of a Derbyshire Practitioner

**Published:** 1807-08

**Authors:** 


### *To the Editors of the Medical and Physical Journal.*

GENTLEMEN,

FROM the perusal of the various papers in your Journal, I have derived much pleasure and great information; and I consider a periodical work of that nature as a vehicle admirably calculated to diffuse, through a large space, the streams of medical knowledge; and by collision of sentiments upon subjects, respecting which there has been a diversity of opinion among practical men, and by fair manly discussion to elicit truth, the great object of all enquiry.

Yet although I confess that I have read, with great satisfaction, papers written in this spirit, and without flattery entertain a high sense of your discrimination respecting the admission of them, yet I cannot restrain my indignation, when I see the purpose of your Journal abused as it is by a miscellaneous paper, occupying three pages of the Number for this month, of which the clear object is to give vent to a splenetic disposition, without conveying one useful useful idea. The letter is divided into three parts, viz. on *infantile diseases*, on a *demonstration of a brain*, and on the *treatment of a case of popliteal aneurism.* I have placed them in an inverted order, according to the real importance of them.

Now, mark the information which he gives on infantile diseases! He says, that "an admired Reporter observes, in your crowded community, (London), the mortar and pestle are hourly chargeable with murder," and then, for your information and that of all your readers, he adds, that in croup, chin-cough, measles, and pneumonia, which are daily carrying off a portion of innocent and highly-regretted little victims, the lancet, if timeously resorted to, the leech, or the purge, are of great use. This is the whole of his observations upon the copious subject of *infantile diseases*, and happy it is for the natives and inhabitants of Derbyshire that they have so wise a practitioner among them. It is a pity that he did not give his name and place of abode, that they might have known where to apply for assistance in these juvenile diseases, at least where not to apply.

Another part of the strictures of the *Derbyshire Practitioner* is on a demonstration of the brain.

What he means should be understood by this part of his paper, I really am entirely at a loss to imagine. The facts are these:

At the last Derby Assizes a man was convicted of murder, and his body given to the surgeons for dissection. The dissection was admirably performed by a gentleman of good abilities, and very conversant with anatomical subjects; and some drawings made by a very ingenious man, not of the medical profession, from some parts, especially the dissection of the parts concerned in the operation for the popliteal aneurism, both in their natural state, and when altered in the operation from their natural position. Many medical men of Derby and its vicinity attended the dissection, and some of them were desirous of seeing the state of the brain in a person destroyed by hanging. It was therefore examined by the gentleman who conducted the dissection, who was very much accustomed to such a task, and by others who were present. No detailed demonstration was attempted or given. These are the facts, out of which the *Derbyshire Practitioner* continues to observe, that he "had some *awkward* feelings; *awkward*, because he did not conceive himself in a situation to interfere." I dare say that he had some awkward feelings of incapacity and inferiority, if if I may judge from the composition of the whole of his paper, at the end of which he mentions the name of Sir William Blizard, whose lectures our Derbyshire Practitioner attended. You, Gentlemen, are well acquainted with the personal worth and professional character of this Gentleman; no man has contributed more in every way to the advancement of his profession; and if all his pupils have not attended to his instructions and profited by them, the fault is not in the teacher.

The other part of the paper of the *Derbyshire Practitioner,* is evidently designed to convey a censure upon the treatment of a case of popliteal aneurism, in the Naval Hospital at Deal. I have again read through the history, treatment, and remarks on that case in your Journal, and it appears to me that from the commencement to the fatal termination of it, nothing could be more judicious.

The surgeon waited before the operation to give time to the anastomising branches to dilate, then performed Mr. Hunter's operation; and upon a hæmorrhage subsequently taking place, which endangered the man's life, amputated the limb as the only chance of saving it.

Yet the *Derbyshire Practitioner* "cannot think that the moment for amputation was properly chosen," when the patient had lost four pounds of blood. I presume that he thinks it would have been better to have waited till he had lost eight. He mentions it as a kind of discovery of his own, "that the operation (of amputation) could not be undertaken without the prospect of losing some;" and further, "that there is a point *we* conceive, in extreme inanition, a single advance beyond which is positive and immediate destruction.

Here, Gentlemen, are two great discoveries made by the *Derbyshire Practitioner.* The first is, that in cutting off a limb some blood may be lost. I am sure that this must surprise you, who live in the metropolis, and often witness such operations. The other is, that when more blood is lost than is compatible with life, a man dies. Can any thing be more astonishing? Yet the Surgeon at the Deal Naval Hospital had the wit to discover this first, and therefore wisely determined to amputate the limb, without being aware that a Practitioner in Derbyshire would make the same discovery.

The *Derbyshire Practitioner,* or *Practitioners,* (for he always expresses himself by the plural *we*) next observes, that *we* have no means of knowing whether the bleeding was venous or arterial, and then, in three lines afterwards, adds, adds, " nor is it perhaps material to the present question." If then, according to his own shewing, it was not material to the present question, where was the use of the inquiry?

Next the *Derbyshire Practitioner* suggests his mode of managing the patient. *We* would first think of means to stay for a while the vital torrent, (" what bombast!!) *We* would have dilated the cyst to its extent; and would then, after evacuating it of its accumulated filth, have crammed it with graduated compresses of sponge, and with the help of a bandage, would have kept up a certain resistance to the bleeding point, or surface." All this *we* would have done. Can more preposterous nonsense be written than this, as applicable to such a case, especially when the surgeon, who treated the patient, states, that " after the most severe pressure which possibly could be made," he lost some blood.

The *Derbyshire Practitioner* next, with great gravity, adds, " the limb, no more than the life of the patient, might not have been saved by such procrastination." So he recommends a practice, by which neither life nor limb might have been saved, and tells his medical brethren that it seems to *us*, as if it would have been the most rational practice.

I intreat your pardon for trespassing thus far on your valuable pages, and intreat that you will in your next Number grant a place to these remarks, from your constant reader,

MOROMASTIX.

